# Disease of Dietetic Origin

**Published:** 1893-12-16

**Authors:** John Beddoe

**Affiliations:** Late Physician to Bristol Royal Infirmary


### The Hospital Clinic.

[*The Editor will be glad to receive offers of co-operation and contributions from members of the profession. All letters should be addressed to* The Editor, The Lodge, Porchester Square, London, W.]

### DISEASE OF DIETETIC ORIGIN.

By John Beddoe, B.D., M.D., F.R.S., late Physician to Bristol Royal Infirmary.

Some of the varying aspects of disease having a dietetic origin are so well exemplified by the following cases, that a brief *résumé* of their clinical aspects and the dietetic and general treatment adopted will illustrate some points of considerable interest to practitioners of medicine.

R. A., a strongly-made labourer, was brought into the Bristol Royal Infirmary perfectly comatose, and with right hemiplegia. He was said to be a single man, living alone in lodgings, and no further history could be obtained. On careful examination sundry bruises or hæmorrhagic discolorations were detected, some of which were in situations where they were unlikely to have arisen from accident; there was also a certain degree of sponginess of the gums. In other respects the man did not appear ill-nourished.

Scurvy was diagnosed; and, as deglutition was not impaired, there was no difficulty in administering lemon-juice and broth containing fresh vegetables. Under this treatment, and the use of aperients, the patient began to amend immediately, and in a very few days had recovered consciousness and a considerable amount of power in the right arm and leg. He was now able to explain to us the cause of his symptoms. He lived alone, as we had been informed, and was in receipt of very fair wages; but, having no one to cook for him, and not caring to take the trouble to cook for himself, he had contracted the habit of subsisting almost entirely upon bread and cheese, with very little meat and no vegetables. On this system his strength had been fairly maintained, nor did he seem to have had any suspicion that anything was going wrong with him until shortly before the occurrence of the hemiplegic attack.

The same treatment was continued, with small doses of iodide of potassium, and the patient continued to improve rapidly. All trace of paralysis disappeared, as completely as did the superficial ecchymoses; and the man was ultimately discharged perfectly well, with careful directions as to the future management of his diet.

2. W. B., soldier, æt. 42, had been living in the West Indies, where his health had been fairly good. Six weeks ago he embarked for England. He states that he and his companions had salt provisions, but no lime-juice; that they had very rough weather and underwent much hardship, and that their vinegar-cask got destroyed and its contents lost during a storm. That about four weeks ago large pustules (ecthymatous or rupial?) began to break out on his legs.

The man came in in wretched condition, emaciated, with many small circular ulcers on the legs, on some of which were rupial crusts. The gums were spongy and sore, with ulceration beginning round the necks of the teeth. Yet there were nowhere any typical scurvy-blotches, only slight discolorations. The complexion was earthy and haggard.

With full diet, plenty of green vegetables and lemon juice, and quinine, the man improved very rapidly, and went out quite well in about a month.

R. H., æt. 35, labourer, a strongly-made, ruddy, healthy man, who had been doing field-work, and living temperately, eating principally potatoes and bread and cheese.

On November 1st, while at work in the field, he was seized with violent pain in the lower part of the abdomen, followed by sickness. His bowels had previously been open regularly, and he had one natural stool on the 1st, after the beginning of the attack. He dropped his work at once; but it was not till the 4th that he sought medical advice. Dr. Grace, of Downend, attended him. He had then much pain in the lower part of the abdomen, and had pretty frequently stercoraceous vomiting; he kept down no food whatever. Dr. Grace administered sundry aperients, large doses of calomel, and large and repeated injections; these having no effect on the symptoms, he sent the man into the infirmary on the 7th.

On his admission, his countenance was rather anxious; the pain was said to be variable, not very severe; the tenderness was slight, but extended over the whole lower part of the abdomen, and was not specially localisable; the left iliac region was rather fuller than the rest of the abdomen, and was more irregular on palpation, but was tolerably resonant on percussion. Tongue foul, greenish brown; pulse 80-90, with nothing very remarkable in its character. Immediately after admission, he had vomited fluid of distinctly fæcal odour. He was able to lie quietly, and to change his position easily.

Passed O'Beirne's tube easily, and injected fully two quarts of warm water; it was retained a good while, but brought away nothing. Administered three successive 5-grain doses of calomel, and gave him a hot bath, all without effect. Food (bread, beef-tea, and tea), was given in small quantities, and turpentine was applied at night to the abdomen, when half a grain of morphia was given, as the pain had increased.

8th. Has vomited a large quantity of dirty greenish-brown fluid, with little or no fæcal smell. Has slept a little. Pulse 72. No other change; but he looks less anxious. Urine very scanty; ℞. Aloes soc. gr. i.; pil. saponis co gr. ii. ss, quartis horis. Abdomen to be kept covered with a thin warm poultice. To take very small quantities of beef tea or wine, with a morsel of bread from time to time.

11th. Has been going on quietly with little change. Has not vomited for two days. Pain much less; countenance no longer anxious; can bear examination very well. Pulse 72.

12th. No change; has not vomited; tongue cleaning fast.

13th. Last night had a large natural evacuation, which passed without much straining, and without any pain. It consisted of soft fæcal masses, with a number of pieces, varying in size up to that of a boy's marble, of a pale softish substance, apparently ill-masticated potato. Abdomen not much altered in shape or resonance by the evacuation.

Medicine was now discontinued; after another day had elapsed he was allowed a plentiful diet; the bowels were opened regularly and naturally every day; and in the course of a week he was discharged.

In this case the seat of obstruction was doubtless somewhere in the ileum. The man's constitution was very favourable, both physically and morally. It will be observed that even before the treatment, which ultimately proved effectual, had begun to act, there was a tendency to the establishment of toleration. The compound of aloes and opium is very generally successful in cases of simple obstruction like this.

